# Screening for Sarcopenia (Physical Frailty) in the COVID-19 Era

**DOI:** 10.1155/2021/5563960

**Published:** 2021-05-21

**Authors:** Amira Mohammed Ali, Hiroshi Kunugi

**Affiliations:** ^1^Department of Behavioral Medicine, National Institute of Mental Health, National Center of Neurology and Psychiatry, Tokyo 187-8553, Japan; ^2^Department of Psychiatric Nursing and Mental Health, Faculty of Nursing, Alexandria University, Alexandria, Egypt; ^3^Department of Psychiatry, Teikyo University School of Medicine, Tokyo, Japan; ^4^Department of Mental Disorder Research, National Institute of Neuroscience, National Center of Neurology and Psychiatry, Tokyo, Japan

## Abstract

Although the numbers of aged populations have risen considerably in the last few decades, the current coronavirus disease 2019 (COVID-19) has revealed an extensive vulnerability among these populations. Sarcopenia is an age-related disorder that increases hospitalization, dependencies, and mortality in older adults. It starts to develop in midlife or even earlier as a result of unbalanced diet/poor nutrition and low levels of physical activity, in addition to chronic disorders such as obesity and diabetes mellitus. Given that social isolation is adopted as the most protective measure against COVID-19, the level of physical activity and the intake of adequate diet have considerably declined, especially among older adults—denoting an increased possibility for developing sarcopenia. Research also shows a higher vulnerability of sarcopenic people to COVID-19 as well as the development of wasting disorders such as sarcopenia and cachexia in a considerable proportion of symptomatic and recovering COVID-19 patients. Muscular wasting in COVID-19 is associated with poor prognosis. Accordingly, early detection and proper management of sarcopenia and wasting conditions in older adults and COVID-19 patients may minimize morbidity and mortality during the current COVID-19 crisis. This review explored different aspects of screening for sarcopenia, stressing their relevance to the detection of altered muscular structure and performance in patients with COVID-19. Current guidelines recommend prior evaluation of muscle strength by simple measures such as grip strength to identify individuals with proven weakness who then would be screened for muscle mass loss. The latter is best measured by MRI and CT. However, due to the high cost and radiation risk entailed by these techniques, other simpler and cheaper techniques such as DXA and ultrasound are given preference. Muscle loss in COVID-19 patients was measured during the acute phase by CT scanning of the pectoralis muscle simultaneously during a routine check for lung fibrosis, which seems to be an efficient evaluation of sarcopenia among those patients with no additional cost. In recovering patients, muscle strength and physical performance have been evaluated by electromyography and traditional tests such as the six-minute walk test. Effective preventive and therapeutic interventions are necessary in order to prevent muscle loss and associated physical decline in COVID-19 patients.

## 1. Introduction

Aging involves deterioration of cellular processes, inability to maintain homeostasis, and increased vulnerability to stressors [1–4]. This condition is known as frailty. Because it embroils accumulation of subclinical declines in physical, psychological, social, and nutritional aspects, frailty is not easy to identify in clinical settings or research [1, 5]. Therefore, researchers commonly focus on the physical aspect of frailty, which is usually referred to as “physical frailty/frailty phenotype” or “sarcopenia.” In fact, both sarcopenia and physical frailty represent an early stage of physical dependence and disability in old age [6]. Sarcopenia is an age-bound condition that entails a progressive loss of skeletal muscle mass along with declines in muscle strength and physical performance [3, 7].

Sarcopenia is reported in around 60% of older adults aged 80 years or above [8–12]. Its prevalence is increasing because of the rising life expectancy. Statistics predict that the number of sarcopenic older adults will double in the next 30 years [13–15]. Likewise, the number of older adults in need for long-term rehabilitation will quadruple by 2050 [12, 16], primarily because of the increased prevalence of musculoskeletal disorders, frailty, sarcopenia, visual and hearing impairments, fatigue, cognitive decline, sleeping disorders, and depression [15–18].

Loss of muscle mass contributes to a countless number of adverse effects including chronic pain (e.g., back pain), increased risk for chronic debilitating disorders (e.g., obesity and type 2 diabetes), frailty, and progressive decline in functional capacity and independence [15, 16, 19, 20]. Old people with muscle weakness have 4.3-fold greater risk for slow gait speed and 2.6-fold greater risk for severe mobility limitation [16]. Physical disability in sarcopenic people is common regardless of ethnicities, health behaviors, fat mass, and comorbidities [21]. The term “sarcopenia with limited mobility” refers to sarcopenic patients in need for therapeutic interventions because of loss of function resulting from skeletal muscle weakness [14]. Sarcopenic individuals are highly prone to falls, hospitalization, and mortality [8, 22]. A recent meta-analysis reports poor overall survival and relapse-free survival among sarcopenic patients with head and neck cancers compared with nonsarcopenic counterparts [22].

The current coronavirus disease-2019 (COVID-19) pandemic has been associated with a remarkable increase in key risk factors associated with sarcopenia such as decreased physical activity and unhealthy eating patterns (e.g., frequent snacking and skipping meals), which are most noticed in older adults [23–26]. On the other hand, sarcopenia is a disorder that entails chronic inflammation, malnutrition, and metabolic and endocrinal dysregulation, as well as several systemic dysfunctions (e.g., motor nerve degeneration) [3, 7]. Moreover, diaphragmatic muscle thickness is considerably reduced in sarcopenic individuals, which may evoke respiratory failure in critically ill patients [15]. Therefore, sarcopenia stands for a key risk factor that heightens the vulnerability of older adults to COVID-19. In fact, older adults represent the vast majority of patients with symptomatic COVID-19, and their prognosis is rather poor [27, 28]. Preexisting sarcopenia is associated with progressing to disease severity in COVID-19 patients reflected by polypharmacy, multiple organ failure, intensive-care unit (ICU) admission, increased need for mechanical ventilation, and mortality [29–34]. Among community-dwelling older adults, muscle mass is an independent correlate of maximum expiratory pressure, an indicator of respiratory muscle strength [35]. Myokines produced by wasting muscle (e.g., interleukin- (IL-) 6 and IL-7) can considerably alter immunity [36].

Cytokine storms associated with COVID-19 infection activate oxidative and catabolic signaling, which accelerate muscle protein degradation leading to skeletal muscle loss [37, 38]. Body protein loss, indicated by higher levels of blood urea nitrogen and reductions in serum total protein and albumin levels, is evident in severe and fatal COVID-19 patients, and it is associated with the development of multiple organ failure and mortality [37]. Significant weight loss is recorded in 61% of recovering COVID-19 patients; in 26.2% of these patients, weight loss was greater than 10% of body weight [39]. Although weight loss is higher in ICU-admitted patients [39, 40], it was also recorded in patients treated at home [41]. Fatigue and myalgia are common symptoms in severe and fatal COVID-19 patients while markers of muscle damage (e.g., creatine kinase, CK) are rather high [3, 28, 37, 38]. Body composition estimation in ICU-admitted COVID-19 patients denotes excessive loss of muscle mass following their ICU stay [42]—highlighting the high vulnerability of these patients to ICU-acquired weakness [38]. Respiratory symptoms are usually more severe in COVID-19 patients expressing muscle injury, which denotes a possibility of respiratory muscle insult [43, 44]. Postmortem analysis of muscle tissues of COVID-19 patients demonstrates massive necrosis and atrophy of myofibers along with myofibril disarray and Z-disc streaming [45]. In the meantime, COVID-19 survivors express marked reductions in hand grip and physical functioning—they suffer mild fatigue and dyspnea while they perform activities of daily living (ADL) [46]. Altogether, skeletal muscle failure seems to be one of the multiple organ failures that strike COVID-19 patients either due to the dystrophic effects of cytokine storms or due to direct viral binding to its angiotensin-converting enzyme 2 (ACE2) receptor on the surface of skeletal myocytes [47, 48]. Because muscle wasting in hospitalized patients is associated with serious adverse effects including premature death [37, 38], researchers emphasize the importance of screening hospitalized COVID-19 patients, especially older adults, for wasting disorders such as sarcopenia and cachexia [3, 45, 49].

Sarcopenia can be mitigated at early stages by physical activity, high-protein diets (especially those rich in milk soluble proteins), and supplements (e.g., amino acids, vitamin D, and polyphenols in bee products) [3, 7, 20]. Meanwhile, protein and amino acid supplementation to COVID-19 patients who are prone to sarcopenia may improve recovery, decrease ICU admission, and probably prevent muscle loss that frequently occur in patients with prolonged disease course [38]. Therefore, early identification of vulnerable groups through proper screening may be necessary to facilitate efforts for the prevention and/or treatment of sarcopenia during the current COVID-19 crisis. Screening for physical frailty in non-COVID-19 patients and in older populations has been integrated in primary healthcare in some countries as an attempt to target immune vulnerability among these individuals during the current outbreak [50–52]. Likewise, identification of COVID-19 patients liable to skeletal muscle injury may be necessary to provide adequate nutritional and physical rehabilitation therapies in order to facilitate recovery and prevent postrecovery disability [28, 38, 46]. Given that routine clinical care for COVID-19 patients focuses primarily on promoting survival, the estimation of muscle quantity or muscle strength is less considered, which may be associated with extreme later suffering after discharge out of the development of disability, even in young individuals [15]. Nonetheless, the novelty of the disease as well as its acute and progressive nature entail lack of knowledge regarding the most effective measures for assessing muscle condition in severe cases. Sarcopenia and similar wasting conditions (e.g., cachexia and malnutrition) are widespread in community-dwelling elders and in a variety of non-COVID-19 patients (e.g., diabetics and obese). However, measures that flag disruptions in muscle strength and muscle quantity are lacking in most primary-care settings, albeit anthropometric measures are conducted in some diabetes clinics [15]. To bridge the gap, this review sheds light on conditions in which the assessment of skeletal muscle damage may be necessary. It also provides a detailed illustration of different measures used for detecting sarcopenia, with a focus on measures appropriate for use among patients struck by COVID-19.

## 2. Relevance of Body Composition to Sarcopenia and COVID-19

Body composition plays a role both in sarcopenia and COVID-19. In particular, high fat mass in obese and overweight older adults is associated with extensive muscle breakdown, which is hidden behind the fat bulk, and therefore, it frequently goes unnoticed [15, 53]. Adipokines such as leptin stimulate an excessive release of cytokines such as interlukin-6, which activate muscle fiber remodeling resulting in alterations in anthropometric measures, even in older people with cancer [54]. The co-occurrence of sarcopenia and obesity is a condition known as sarcopenic obesity [3]. Renal injury is common in COVID-19 patients even after recovery [55]. Longitudinal data show increased risk for renal dysfunction in sarcopenic and obese people [56]. These patients also demonstrate poor respiratory muscle performance, which is not frequently checked in routine care even for people with symptoms such as dyspnea, and it is associated with respiratory dysfunction in these individuals when they contract COVID-19 [15, 35, 57].

Both general and central obesity are associated with a high risk for COVID-19 infection [58]. Body mass index (BMI) is higher in severe COVID-19 patients (mean difference = 1.6, 95% confidence interval (CI): 0.8–2.4; *p* = 0.0002, *I*^2^ = 75%) [59]. Indeed, obesity is the most prevalent chronic comorbidity among symptomatic COVID-19 patients (42%, 95% CI: 34–49%, *p* = 0.034, *I*^2^ = 69.2%) followed by hypertension (40%, 95% CI: 35–45%, *p* = 0.001, *I*^2^ = 55.6%) and type 2 diabetes (17%, 95% CI: 15–20%, *p* = 0.001, *I*^2^ = 32.6%) [60]. Among different comorbidities, COVID-19 patients who are obese express the highest overall pooled event rate for severe complications (56.2%, 95% CI: 35.3–75.1, *p* = 0.015, *I*^2^ = 71.5%) [61]. Several meta-analytic reviews associate BMI ≥30 kg/m^2^ in COVID-19 patients with composite poor outcome, which consist of hospital admission, ICU admission, ARDS, severe COVID-19, higher use of mechanical ventilation, and mortality. Odds ratio reported in these studies range between 1.4 and 2.0 (*p* values <0.001) [59, 60, 62], with consistency of the findings across different primary studies (*I*^2^ = 0%) [59].

## 3. Identification of Individuals with/or Prone to Sarcopenia (Target Groups)

Loss of muscle mass and associated muscle weakness can be reverted, especially during early stages, through sound exercise and nutritional interventions [19, 63–69]. Evidence from animal studies shows that nutritional interventions for muscle wasting yield better results when administered early during the development of muscle atrophy [7, 70]. Therefore, proper screening and management of sarcopenia may support efforts aimed to slow or reverse the progression of physical frailty in old seniors [1]. Indeed, the latest available guidelines for frailty management strongly recommend screening older adults for physical frailty and its underlying causes via rigorous tools, treating sarcopenia as a main cause of weight loss, and addressing nutritional needs in this group [14, 71]. In the same way, current guidelines emphasize the need for assessing sarcopenia and physical frailty in patients with COVID-19 who get hospitalized [30, 31, 33, 72]. Screening for sarcopenia and physical frailty has the merit of identifying potentially remedial conditions while enjoying a noninvasive nature [73]. Thus, timely identification of skeletal muscle wasting in people with potentially modifiable risk factors is of indispensable importance for a successful development of interventional strategies that can restore motor functioning and prevent disability in older individuals and in COVID-19 patients [11, 74].

Sarcopenia has a multifaceted dynamic that entails undernutrition, inflammaging, oxidative stress, motor neuron injury, satellite stem cell dysfunction, metabolic alterations, decreased physical activity, and dysregulation of gut microbiota [3, 7]. Frailty is closely linked to comorbidity, poor cognition, functional dependence, institutionalization, and hospitalization [3, 75]. Therefore, it is recommended to screen for sarcopenia in people with recent functional decline, recent history of reduced appetite that resulted in poor food intake, unintentional weight loss of more than 5% per month, low muscle mass, repeated falls, depression, cognitive decline, and chronic wasting disorders such as chronic heart failure, chronic obstructive pulmonary disease, diabetes mellitus, chronic kidney disease, connective tissue disease, and tuberculosis [14, 76, 77]. Patients with chronic illnesses who undergo surgery may also be screened for sarcopenia both pre- and postoperatively. Sarcopenia is associated with malignancies, and it contributes to greater complications and death postoperatively in these patients [78, 79].

The literature documents sex differences in the onset of sarcopenia. Aging of skeletal muscle develops earlier and faster in women than in men [80–82]. The drop of estrogen, which starts as early as the age of 45 years, is a direct read out for senescence in women, and it promotes the development of several age-related pathologies, including sarcopenia [2, 4, 18]. Estrogen deficiency alters glucose and lipid metabolism, fosters skeletal muscle apoptosis, and hinders processes involved in muscular force generating capacity such as myosin phosphorylation and satellite cell function, which are highly sensitive to estrogen [20, 83]. Evidence reveals greater age-related dysfunction in myosin and myosin ATP turnover in females than in males [82]. A recent meta-analytic review shows that higher levels of plasma IL-6 are associated with worse changes in muscle quantity and quality in aged women than in aged men probably due to the protective role of testosterone [80]. Among hospitalized older adults with COVID-19, frailty is higher in women than in men (75.2% vs. 59.4%, *p* < 0.001) [84]. DNA methylation of genes that regulate energy metabolism and oxidative stress is more evident in myoblasts and myofibers of women than in those of men [81]. Sarcopenia evaluated by criteria based on the percentage of skeletal muscle mass is associated with three single-nucleotide polymorphisms (SNPs) in women: FTO rs9939609, ESR1 rs4870044, and NOS3 rs1799983 [85]. Therefore, women demonstrating these SNPs as well as menopausal women and young women with ovulation failure should be addressed as candidate targets for the prevention and treatment of sarcopenia.

COVID-19 patients either treated at home or in the hospital, especially in the ICU, are prone to wasting and muscle loss [39, 41]—in particular, patients with polypharmacy, prolonged hospital stay, immobility (in the prone position for a long time), body protein loss, and gastrointestinal symptoms that decrease food intake or increase nutrient loss (anorexia, vomiting, and diarrhea) [28, 37, 38]. Therefore, skeletal muscle assessment may be necessary in severe patients exhibiting weight loss and malnutrition or a high risk for malnutrition [28, 38].

## 4. How to Screen for Sarcopenia

To screen for sarcopenia in research and clinical settings, researchers have developed a wide range of predictive risk models, which consider numerous variables such as age, sex, race, and comorbidities [86, 87]. The main muscle-related elements that should be evaluated in sarcopenia are the amount of muscle (mass) and its ability to generate force (strength) or function properly (physical performance) [21, 76]. Beyond these measures, current guidelines recommend interventional studies addressing sarcopenia to assess a number of outcome indicators that take into account the progressive nature of sarcopenia [16, 76].

**Figure 1:**
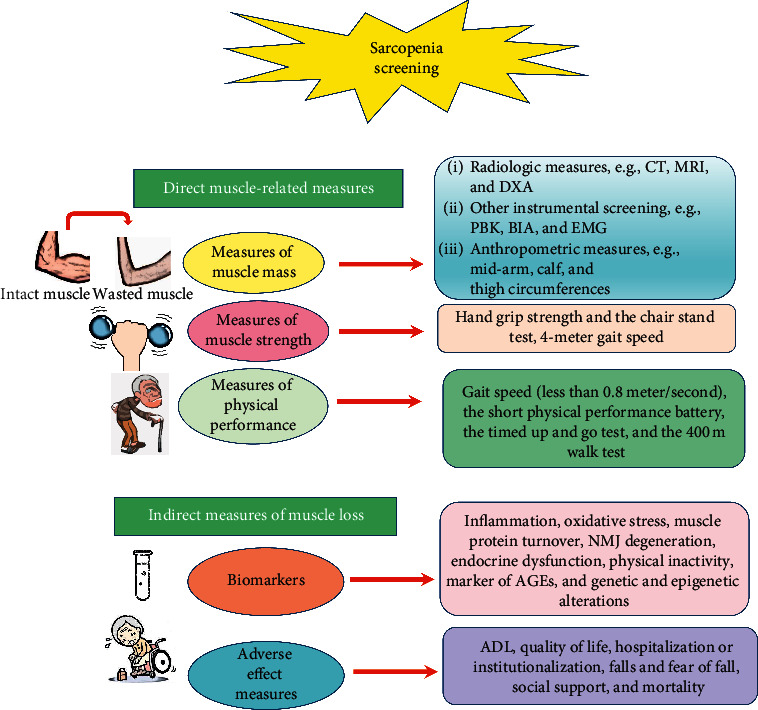
Schematic illustration of the algorithm used for sarcopenia screening. Abbreviations: CT: computed tomography, MRI: magnetic resonance imaging, DXA: dual-energy X-ray absorptiometry, PBK: partial body potassium, BIA: bioelectrical impedance analysis, EMG: electromyography, and NMJ: neuromuscular junction. The most commonly used algorithm for the diagnosis of sarcopenia involves direct assessment of muscle strength and physical performance using gait speed and handgrip strength. When the evaluation reveals muscle weakness and poor muscle function, direct estimation of muscle mass via CT, MRI, or DXA is necessary to confirm the diagnosis. However, muscle mass can be evaluated by cheaper and less sophisticated techniques such as BIA and even by anthropometric measures when other techniques are not available. A variety of indirect measures of muscle quality can be employed in research and clinical settings in order to evaluate the effectiveness of therapeutic protocols that target sarcopenia. Pathologies underlying muscle failure as well as response of sarcopenic muscle tissues to treatment can be detected via a wide range of biomarkers. In addition, numerous health-related outcomes that affect sarcopenic people can be used as indicators of treatment effectiveness, e.g., falls and hospitalization.

Based on the recommendations declared by the Asian Working Group for Sarcopenia (AWGS), European Working Group on Sarcopenia in Older People (EWGSOP), and many others [21, 76, 88], we attempted to summarize screening measures that can uncover aspects of failure in skeletal muscle quantity and quality (Figure 1). These measures were broadly categorized into two groups: direct and indirect measures of muscle loss. The former group represents primary indices that directly reflect qualities of muscular mass, strength, and function/physical performance [21, 76]. They can also portray overtime changes that result from muscle fiber transformation in response to aging and environmental factors [16, 76]. The second group comprises a large set of secondary parameters, some of which can speak for age-related molecular and cellular changes taking place in skeletal muscle (e.g., various biomarkers), and some others reflect a trail of sarcopenia-related adverse effects, which take physical, cognitive, emotional, and social forms [8, 76, 89, 90].

### 4.1. Direct Measures of Sarcopenia

#### 4.1.1. Measures of Skeletal Muscle Mass

Skeletal muscle mass can reflect changes in quality of life (QoL) [91]. A number of body-imaging techniques can provide data necessary to estimate muscle mass [76, 87, 92] (Table 1). The five key aspects of sarcopenia evaluation that are addressed by imaging techniques include the thickness, cross-sectional area, echogenicity, fascicle length, and pennation angle of the addressed muscle segments [93, 94]. The literature reports wide variations in the rates of sarcopenia owing to limitations intrinsic in its assessment tools as well as heterogeneity of the assessed populations (e.g., critical patients, geriatric patients, and community-dwelling elderly) [95–97]. Moreover, proper measurement of muscle amount and strength is rather challenging given that the available assessment techniques demonstrate a range of advantages/disadvantages with regard to their reliability of measurement, cost, safety, availability, and ease of use [21, 87, 92]. Magnetic resonance imaging (MRI) and computed tomography (CT) are considered gold-standard techniques for estimating body composition, including muscle density and fat infiltration. They are preferably applied to certain muscles, but they can also scan the whole body [94, 98].

MRI produces high-resolution images that detect muscle, fat mass, and water contents of the body based on the different molecular properties of these anatomical compartments. It simultaneously detects qualitative abnormalities such as muscle disruption, edema, muscle-fat infiltration, and fibrosis [98]. Technically, MRI identifies variations in body structures by detecting radiofrequency signals (T1 and T2) emitting from nuclear spinning towards the direction of an external magnetic field. Mid-thigh or the abdomen at lumbar level mid-L3 are the key areas used to reflect on muscular condition of the whole body. Intermuscular and intramyocellular lipid depots are noted by a short T1 and a long T2 proton relaxation time [99]. A systematic review reports good-to-excellent manual slice-by-slice segmentation reliability in eight studies and moderate-to-good validity against dissection in one study, while the validity of automatic techniques combined with different statistical shape or Atlas-/image-based methods was good in four studies. Manual segmentation is used as a gold-standard method for muscle quantification; however, it is associated with greater errors in volume and shape estimations [96]. MRI types vary based on their molecular basis and scanning techniques such as 23 Na MRI (based on skeletal muscle tissue sodium concentration, TSC), CT muscle attenuation, diffusion tensor MRI, Dixon MRI, and proton magnetic resonance spectroscopy (MRS) (e.g., 13C and 31P) [93, 94, 100]. MRI has high repeatability [100]. It assesses fat infiltration, edema, and specific patterns of muscle involvement. MRI measurements are reported to correlate with disease severity in spinal bulbar muscular atrophy and amyotrophic lateral sclerosis, two key disorders of motor neuron loss [101]. MRI involves no radiation risk, but it is expensive, and it requires technical skills and space, which make its use limited to research facilities [99]. The reproducibility and concordance of abdominal skeletal muscle area segmentation analyses by CT and T2-weighted (T2w) MRI abdomen/pelvis are documented as a measure of sarcopenia in renal cell carcinoma patients, denoting that these measures may be used interchangeably [78].

Several studies report reductions in skeletal muscle mass measured by CT in hospital-admitted and critical patients with various health problems, denoting a significant association of muscle mass loss with mortality within one month after hospital admission [102, 103]. Some indices are even based on the quantification of the mass of certain muscles by CT such as the psoas muscle index, which is based on the quantification of the psoas muscle located at the base of the fourth lumbar vertebra. This index is a reported predictor of morbidity, length of hospital stay, and in-hospital complications such as mortality in the elderly and in trauma population [103, 111]. CT effectively identified muscle mass loss in COVID-19 patients over the course of ICU stay [42]. CT may simultaneously measure muscle mass in patients using it for other purposes, e.g., in cancer patients who frequently have this procedure as a regular part of their standard management [79]. CT is used to assess the extent of lung fibrosis in COVID-19 patients. The cross-sectional areas of the pectoralis muscles (PMA, cm^2^) can be automatically measured on axial CT images. Pectoralis muscle index (PMI) is calculated as: PMI = PMA/patient's height square (m^2^). PMI significantly predicted the length of hospital stay, intubation, and mortality in COVID-19 patients [57]. Sarcopenia assessed by PMI is an independent risk factor for mortality in patients with lung cancer [112]. Cumulative literature shows significant variations in CT techniques regarding contrast use, selected skeletal muscle areas, ranges of radiodensity delimitation, and their cutoff points. A key reason for such variations is lack of precise information about the correlation between skeletal muscle radiodensity by CT and its molecular composition. Nomenclature uniformization may be established by CT studies that involve direct measures of muscle composition such as MRI [97].

Muscle density can be measured by simpler, safer, and cheaper techniques. For example, dual-energy X-ray absorptiometry (DXA) is a relatively safe technique that entails low radiation exposure [21, 76, 105, 110]. DXA scans the body depending on variations in the absorption of low- and high-energy X-rays by different body components [93, 99]. It is the most frequently used imaging technique for muscle mass estimation. It estimates appendicular lean mass (ALM), the sum of lean mass at the upper and lower limbs. ALM is used to calculate appendicular lean mass index (ALMI = ALM/height^2^) [98]. It also assesses body mineral content and fat mass [98, 113], which make it the most favorable technique for assessing osteosarcopenic obesity—a combination of osteoporosis, skeletal muscle loss, and obesity [3, 113]. DXA is reported to quantify changes in body composition (fat mass and muscle mass) faster and at a greater precision than nuclear MRI [104]. Indeed, the latest consensus report of the EWGSOP recommends DXA as the technique of choice for the clinical assessment of muscle wasting [114]. Despite these merits, DXA has some limitations: the machine used is not portable, DXA scanners are not available in most primary-care settings, various protocols associated with various hardware and software packages between manufacturers make the comparison of results from different settings impractical, DXA does not detect qualitative changes in muscles (e.g., fat infiltration), it is confounded by body size and hydration state because it does not differentiate between water and lean tissue, and it should be avoided during pregnancy [99].

Ultrasound is a cheap, portable, and noninvasive technique that has a high reproducibility. It is also safe because it does not use ionizing radiation [94]. It quantifies tissue thickness based on the detection of an echo reflected back from tissues exposed to ultrasound beams from a transducer [99]. Total quadriceps volume measured by the ultrasound-derived rectus femoris cross-sectional area is a simple index of muscle size. Its high correlation with MRI pinpoints its high reliability [105]. However, lack of standardization, in terms of the most representative muscle sites, and its high dependence on the expertise and skills of the operator are major drawbacks [93]. Moreover, the interpretation of muscle-fat interfaces is limited due to similar acoustic impedance of muscle and fat tissues. In addition, applying the transducer to the skin with excessive pressure may compress the muscle, which may cause measurement errors [99].

The amount of contractile tissues can be quantified via bioelectrical impedance analysis (BIA), a nonimaging tool that quantifies total muscle mass based on the conduction of an electric current applied across the body [98]. BIA is considered a cheap and easy-to-use alternative of DXA [21]. A current systematic review shows that sarcopenia entails low BIA and both are independent predictors of survival in aging samples. However, the extent to which BIA may be valuable in detecting low muscle quality and/or identifying sarcopenia is not clear [115]. Comparisons between CT and BIA in critically ill patients show higher values of skeletal muscle mass than that calculated by CT, especially among males and patients with mild edema [106]. Similarly, comparisons between CT and BIA among geriatric inpatients based on the EWGSOP case-finding algorithm show systematic overestimation of muscle mass by BIA [116]. BIA is considered a proxy of water distribution [94, 115]; however, it may not accurately reflect on body cell mass, especially in critical patients [106, 116]. In addition, BIA is not a standardized technique because BIA equations and cutoff values are population and device specific. Therefore, the prevalence rates of sarcopenia assessed by BIA vary according to equations and devices used for their estimation [95].

Electromyography (EMG) is a nonimaging, noninvasive technique that may evaluate neuromuscular transmission, the degree of denervation indicated by the number or size of motor units and myocytes, and integrity motor units (captured by near-fiber jiggle and jitter), along with edema and deposition of endomysial connective tissue and fat [99, 117]. EMG involves applying a low-intensity (50-kHz) alternating current to the skin to evoke a surface voltage pattern while certain electrodes placed on the sites of muscle of interest monitor the electrical activity of muscle contraction [99, 105, 118]. It revealed significant differences in maximal voluntary strength and motor stability in older adults with presarcopenia, sarcopenia, and severe sarcopenia. However, it detected no differences in the number of motor units in these groups [117]. It also reflected on muscle fiber loss in seven myositis patients similar to diffusion-weighted MRI [119]. An available systematic review indicates that surface electromyography reliably estimates muscle fiber conduction velocity across multiple sessions in sport science, rehabilitation, physiological, and clinical studies [107].

Because 60% of the potassium content of the body exists in skeletal muscle, the estimation of total-body potassium (TBK) has been used as a cheap and safe nonimaging technique that indirectly measures muscle mass [120]. Given that the isotope ^40^K exists at a known and constant natural abundance of 0.0012%, a scintillation counter can effectively measure ^40^K [99]. Therefore, the technique operates by detecting isotropic emissions of gamma rays resulting from the decay of ^40^K, which occurs at a rate of ∼200 gamma rays per second per gram of natural K [99, 109]. Estimations of muscle mass by TBK yielded results consistent with those estimated by DXA among healthy men and women aged 60 years or above [108]. The partial body potassium (PBK) system is a simpler alternative to TBK. It requires subjects to put their arms or legs inside a cavity that comprises a gamma ray detector (4*π* liquid scintillation counter) for 15 minutes [109]. Despite their simplicity and low cost, TBK and PBK are not favorable for estimating muscle mass because muscle mass calculations are based on many assumptions that may not hold in aged or diseased individuals such as constant intracellular potassium content, nitrogen content, and hydration coefficient of lean body mass [99, 120]. In this regard, this technique may not suit COVID-19 patients because they exhibit hypoproteinemia, multiple micronutrient deficiencies, and electrolyte imbalance [28, 37].

Techniques commonly used for muscle quantification (both imaging and nonimaging) are not universally available, especially in low-resource communities, due to their high cost and related technical complexity [21, 92]. Therefore, a number of studies validated the ability of simple anthropometric measures to quantify muscle size and to reliably reflect physical performance and predict survival in old people when sophisticated measures of sarcopenia assessment are not available [21, 87, 110]. These measures are based on the notion that muscle mass of the lower limb is strongly linked to the level of functional impairment in old age [14]. Vastus lateralis, one of the muscle groups of the quadriceps in the thigh that is primarily type II fibers, is most vulnerable to shift to oxidative metabolism, fibrosis, and atrophy with advanced age. Meanwhile, the soleus muscle in the calf largely comprises type I fibers, which naturally undergo higher protein turnover [15, 121, 122]. Low appendicular skeletal muscle mass (ASM) in older individuals could be examined as accurate as relative skeletal muscle index (RSMI) obtained by DXA imaging through simple anthropometric measures such as thigh circumference, calf circumference, mid-arm muscle circumference (MAMC), and total skeletal muscle mass estimated by Lee's formula (eTSMM) [21, 87, 110]. For proper evaluation, these measures should be conducted with a contextual regard for sex, race, and age [87]. Cutoff points for low ASM adjusted for body mass index are <0.789 and <0.512 for men and women, respectively [88]. These cutoffs slightly vary according to ethnicities (e.g., between Asian and Caucasian), body size, life style, and culture [76].

Several models can portray muscle wasting. ASM comprises the mass of the extremities (e.g., measured by DXA) after excluding the mass of bone and fat. The latter is automatically excluded by DXA [91]. According to FNIH guidelines, ASM less than 19.75 kg and 15.02 kg or alternatively ASM adjusted for body mass index less than 0.789 and 0.512 can diagnose sarcopenia in men and women, respectively [16]. Skeletal muscle mass index (SMI) is a common measure of muscle mass. It can be calculated by dividing the skeletal muscle mass of the upper and lower limbs by height squared [8]. ASM/height^2^ less than 7.0 kg/m^2^ and 5.5 kg/m^2^ represent recommended cutoff points for diagnosing sarcopenia in men and women, respectively [12, 76]. Moon et al. diagnosed sarcopenia via a novel index based on lower extremity skeletal muscle mass (LESM, excluding mass of fat and bone). This index is calculated by dividing LESM by lower extremity body weight [kg] × 100 or squared height (LESM [kg]/Ht^2^ [m]) [91].

#### 4.1.2. Measures of Skeletal Muscle Strength

Measures of muscle mass do not consider contraction potential and muscle power generation. Loss of muscle force output is a result of multiple alterations in muscle composition such as fat infiltration and decreased motor units and neural activity, as well as decreased quality of contractile fibers. In fact, dysfunctional or denervated fibers (which are unable to generate force) count; they contribute to muscle mass when assessed by size quantification measures [123]. Functional techniques can express the ability of a muscle to recruit fibers incorporated in motor unit arrays by capturing the kinematic and/or kinetic output exerted during a dynamic muscle action [123, 124]. However, a recent meta-analysis reports no gold-standard technique [124].

Physiological cross-sectional area (PSCA) is a reliable composite measure of the strength and change in strength of leg extension [125]. It is one of the measures involved in the calculation of “relative muscle strength”—a measure of muscle quality that combines muscle size and strength. Relative strength refers to muscle force generation relative to muscle or body size. It is a sound evaluation tool of architectural and functional characteristics of skeletal muscle. However, PSCA is not easy to measure since it requires an apparatus that might not be easily incorporated in clinical settings [123]. On the other hand, muscle strength is frequently measured by reduced hand grip strength for age and gender, the chair stand test, and 4-meter gait (walking) speed [12, 14, 110, 126]. Hand grip to leg extension strength strongly correlates with gait speed, and both are reported to be equally suitable for screening elders for muscle weakness [127]. Cutoff points commonly used to diagnose sarcopenia are usual gait speed less than 0.8 meter/second and hand grip strength less than 26 kg for men and less than 18 kg for women [16, 76]. It is worth noting that measurement of muscle strength may be confounded by non-muscle-related factors such as levels of cognitive function and motivation [21]. Strength measures such as grip strength correlate with functional mobility and incident mobility impairment [123]. Moreover, strength measures positively respond to various interventions for sarcopenia in old people with evident muscle weakness, even with the persistence of low muscle mass [123, 128].

#### 4.1.3. Measures of Muscle Functioning/Performance

Measures of muscle strength and function are interrelated. Physical performance can be evaluated by usual gait speed (less than 0.8 meter/second), the 6-min walk test, 400 m walk, the Timed Up and Go test, the stair climb power test, and the short physical performance battery [1, 14, 21, 110]. The latter evaluates gait, balance, strength, and endurance. It comprises several tests, e.g., standing with feet together in side-by-side, semitandem, and tandem positions; time to walk 8 feet, and time to rise from a chair and return to the seated position 5 times [1, 21].

According to the algorithm set by the European Geriatric Medical Society (EUGMS), Consensus Committee of defining sarcopenia, EWGSOP, and AWGS, the flow of the screening process starts by assessing muscle strength and physical performance using tests of gait speed and handgrip strength. If initial screening uncovers muscle weakness and poor muscle function, muscle mass should be estimated in order to confirm the diagnosis [1, 14, 21, 76].

### 4.2. Indirect Measures of Sarcopenia

#### 4.2.1. Biomarkers

Researchers identified numerous biomarkers for early detection of both sarcopenia and physical frailty as well as for a detailed identification of their main pathophysiological mechanisms, which take place at molecular and cellular bases [110, 122, 129]. For instance, The International Conference on Frailty and Sarcopenia Research (ICFSR) Task Force has recently declared that measuring creatinine excretion via the D3-creatine dilution (D3Cr) method is a more reliable biochemical measure of functional muscle mass than assessment by DXA [129]. Judgment about therapeutic strategies for sarcopenia as either promising or not can be made after a relatively shorter time when treatment effects are assessed at a molecular level rather than at a behavioral level. For example, skin autofluorescence, a marker of advanced glycation end products (AGEs), represents an independent determinant for SMI, hand grip strength, and knee extension strength in older individuals [8]. If an intervention manages to decrease glycation stress at a relatively early stage of treatment, it could be easy to predict improvements in muscle status after an expected duration. Therefore, trials recruiting sarcopenic subjects are recommended to include various biomarkers as indicators of effectiveness [16, 76, 130].

Possible markers of sarcopenia include biomarkers of inflammation and oxidative stress (e.g., interleukin- (IL-) 6, IL-1, tumor necrosis factor-*α*, butyryl-cholinesterase, isoprostanes, oxidized low-density lipoprotein, and vitamins C and E), muscle protein turnover (e.g., creatinine and sarcomeric proteins such as actin, myosin, troponin, and tropomyosin), neuromuscular junction (NMJ) degeneration (e.g., C-terminal again fragment, CAF), endocrine dysfunction (testosterone, DHEA, and GH–IGF-1), growth factors (e.g., transforming growth factor-*β*, myostatin, and activin A and B), physical inactivity (e.g., complement protein C1q, hemoglobin, albumin, leptin, and uric acid), and glycation stress (e.g., skin autofluorescence) [1, 8, 131, 132]. In addition, epigenetic biomarkers of aging (also known as epigenetic clocks) may play an indispensable role in the evaluation of muscle strength and physical performance during old age and their change across various treatment strategies [90]. Sound treatments may induce changes in sarcopenia signature such as the expression level of genes that regulate pre-mRNA splicing, localization, and modification of RNA such as galectin-1, glutamine transporter SLC38A1, and membrane-bound transcription factor protease S2P [89].

#### 4.2.2. Other Measures

The AWGS suggests a range of secondary outcomes to be assessed by interventional studies that address sarcopenia [76]. Sarcopenia is progressive in nature, which implies deterioration of overall health status, e.g., developing back pain (especially when atrophy affects back muscle) and physical dependence, which entails inability to perform ADL. Such drawbacks can seriously alter QoL of sarcopenic patients [1, 11, 14, 19, 53, 90]. Therefore, parameters recommended by the AWGS involve evaluating the dynamic changes in frailty status, basic and instrumental ADL over a given period of time, QoL, and social support. In addition, a number of adverse effects associated with sarcopenia can be assessed such as hospitalization or institutionalization, falls, fear of fall, and mortality [76].

### 4.3. Sarcopenia Assessment in COVID-19 Patients

Despite the current lack of agreed-upon treatment pathways of COVID-19, proposed guidelines highlight the importance of an integrative management, which includes defining and monitoring conditions that heighten immune dysregulation and lead to poor clinical outcomes in COVID-19 patients, such as the nutritional status and body composition, particularly muscle condition [42, 133, 134].

Several studies used clinical measures for the assessment of frailty in COVID-19 patients such as the FRAIL scale [31], Frailty Index (FI) [32], and Clinical Frailty Scale (CFS) [29, 30, 33, 135] to stratify COVID-19 patients according to their need for ICU admission [32], mechanical ventilation, and prolonged hospital stay [33], as well as developing multiple organ failure [31, 33] and mortality [29, 30, 135]. Indeed, a scoping review shows that CFS—the most widely used measure of frailty in COVID-19 patients—is predictive for comorbidity, mortality, complications, length of stay, falls, poor cognition, and functional dependence in 87%, 73%, 100%, 75%, 71%, 94%, and 91%, respectively, of hospitalized older patients [75]. Nonetheless, comorbidities are strong independent predictors for poor prognosis in COVID-19 patients [5, 27, 28]. Around half the items on most frailty assessment tools incorporate the assessment of comorbidities [5, 31–33]. Comorbidity, the accumulation of clinically manifest diseases, is another distinct construct, which represents one etiology of frailty, and it is likely to confound the association of poor skeletal muscle condition with COVID-19 prognosis [5].

Measures of physical frailty are interwoven with those of sarcopenia, which is considered a part and a definite biological basis of the frailty spectrum [1]. Nevertheless, frailty measures mainly address functional independence, which mirrors the physical performance aspect of skeletal muscle quality [33, 75]. However, physical performance may not be seriously altered despite muscle wasting/weakness [7]. Therefore, measures of frailty may be unfavorable for assessing skeletal muscle injury, which is the result of excessive protein degradation that leads primarily to muscle loss/weakness in hospitalized COVID-19 patients, usually over short periods of time [3, 28, 37, 38]. This notion gets support from the findings of a large-scale study predicting COVID-19 prognosis based on two measures of frailty: (1) FI, a 49-item scale that describes aspects of health, disease, disability, and mental wellbeing and (2) the frailty phenotype defined by Fried et al. based on five criteria: weight loss, exhaustion, physical activity, walking speed, and grip strength [49]. Analyses adjusted for sociodemographic and lifestyle factors revealed a higher risk for COVID-19 in prefrail (risk ratio (RR) = 1.47, 95% CI: 1.26–1.71) and frail (RR = 2.66, 95% CI: 2.04–3.47) individuals defined by FI compared to those classified as having robust frailty evaluated based on the criteria of the frailty phenotype [49].

CT can be effectively used to evaluate muscle composition change over the course of hospitalization in severe COVID-19 patients [42]. It may facilitate the detection of frailty among those patients by evaluating PMA [57]. However, the adverse effects associated with the exposure of patients to the ionizing radiation of CT are not known. As shown in Table 1, ultrasound and DXA may be faster and safer alternatives of CT taking into consideration that these measures may be confounded by the patient's body size, weight, and hydration status [99]. Maximal voluntary contraction for quadriceps and biceps was measured by electromyography in COVID-19 patients recovering from pneumonia who had no locomotor disability before contracting COVID-19 [46]. Meanwhile, the use of BIA as a cheaper alternative of CT to diagnose physical frailty in hospitalized COVID-19 elders may not be favorable, at least after the first week of hospitalization. Evidence shows that BIA measures in hospitalized geriatric patients are most reliable during the first week of hospital admission. However, older adults with prolonged ICU stay develop hydration abnormalities, which alter the credibility of this diagnostic technique [133].

Although various biomarkers can be used to signify muscle pathology, several lines of evidence show that the evaluation of CK as a biomarker of muscle injury may be a quick and cheap method to signify the development of muscle atrophy in hospitalized COVID-19 patients [134, 136–140]. The levels of lactate dehydrogenase (LDH) also get altered in severe COVID-19 patients, and they predict poor prognosis [136, 138, 141]. A meta-analysis reports significant differences in LDH between severe and nonsevere COVID-19 patients while changes in CK and other muscle biomarkers (e.g., troponin I and myoglobin) were not significant [141]. However, severe COVID-19 patients, especially those who develop rhabdomyolysis, express exponential alterations in CK [134, 140, 142, 143] and myoglobin [143], which denotes the importance of assessing these parameters in COVID-19 patients who are emaciated or experiencing myalgia.

In addition, grip strength and other indicators of muscle strength and physical performance (e.g., gait speed) are suggested to be used as cheap and quick methods to identify frail people with high risk for adverse effects during the COVID-19 crisis [72]. In particular, global measures of maximal strength of respiratory muscles (e.g., maximal inspiratory pressure) are inversely associated with grip strength. In addition, grip strength can effectively predict disability, morbidity, and mortality in older adults as well as in middle-aged and young people [72]. Indeed, the 6-minute walk test, Chester step test for predicting maximal oxygen uptake (VO_2_ max), spirometry, cardiopulmonary exercise test (for predicting integrative responses of the respiratory, cardiovascular and skeletal muscle systems), and musculoskeletal testing (endurance testing (e.g., push up test) and proximal and distal muscle strength tests (e.g., rising from a squatting position or stepping onto a chair and walking on the heels and on tiptoes)) have been used to detect functional impairments in recovering SARS and COVID-19 patients. Such impairments were associated with reduced QoL, particularly perceived role-physical [55, 144]. Likewise, the number of chair rises in the one-minute sit-to-stand test was associated with reduced strength of the quadriceps and biceps in remitting COVID-19 patients [46].

**Figure 2:**
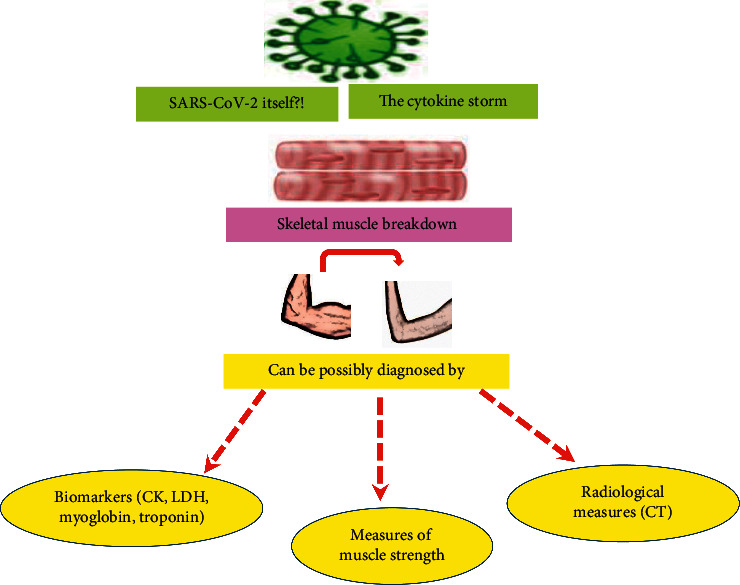
Possible approaches to detect muscle injury in symptomatic COVID-19 patients. Abbreviations: SARS-CoV-2: severe acute respiratory syndrome coronavirus-2, CK: creatine kinase, LDH: lactate dehydrogenase, CT: computed tomography.

Muscle weakness and poor physical performance usually result from muscle dystrophy; measures of muscle strength and physical performance in COVID-19 were used in recovering patients following discharge [46]. Because rapid deteriorations affect severe COVID-19 patients during the acute phase of the disease (e.g., general wasting and malnutrition), imaging techniques (e.g., CT) and laboratory biomarkers may be more desirable for early detection of frail cases than muscle strength measures.  Figure 2 summarizes possible measures that may detect skeletal muscle failure in COVID-19 patients.

## 5. Conclusions

Sarcopenia and related conditions (e.g., osteosarcopenic obesity) are widespread in aged and diseased populations; they are major risk factors for SARS-COV-2 infection. They contribute to the severity of COVID-19 by potentiating cytokine storms and respiratory failure. The detection of sarcopenia in vulnerable groups, mainly older adults and persons with chronic noncommunicable diseases, should proceed from the evaluation of muscle strength by simple measures such as grip strength to the evaluation of muscle mass in those with proven weakness. Muscle mass loss may be best detected by MRI and CT. However, due to the high cost and radiation risk entailed by these techniques, other simpler and cheaper techniques such as DXA and ultrasound are given preference. CT and electromyography were used to evaluate muscle loss in COVID-19 patients. During the acute phase, measuring the Pectoralis muscle mass can be simultaneously performed when lung fibrosis is routinely checked by CT, which entails an efficient evaluation of sarcopenia among those patients with no additional cost. In recovering patients, muscle strength and physical performance have been evaluated by electromyography and traditional tests such as the 6-minute walk test. Effective preventive and therapeutic interventions are necessary in order to prevent muscle loss and associated physical decline in COVID-19 patients.

## Figures and Tables

**Table 1:** Advantages and possible limitations of different techniques used for assessing sarcopenia and the likelihood for their application in COVID-19 patients.

Muscle assessment techniques	Advantages	Limitations	Relevance to COVID-19	Reference
Computed tomography (CT)	It reliably measures muscle quantity and fat infiltration. It can be applied to the whole body or specific muscles and serves as a base for several reliable muscle mass indices. CT routinely used for other purposes (patients with tumors) can simultaneously measure muscle mass. It predicts mortality in critical patients.	Relatively expensive and time consuming, with high radiation risk. No cutoff values for diagnosing sarcopenia.	It has successfully identified muscle loss in ICU-admitted COVID-19 patients screened for lung fibrosis, albeit patients still encounter radiation risk.	[42, 94, 102, 103]

Magnetic resonance imaging (MRI)	It reliably measures muscle quantity and quality (e.g., fat infiltration). It has no radiation risk, and it can be applied to the whole body or specific muscles.	Expensive and not portable or widely available, with technical difficulties and space requirement. There are no cutoff values for diagnosing sarcopenia. Its use is limited to research facilities.	Despite its high accuracy, its use may be impractical because of its limitations.	[94, 99]

Dual-energy X-ray absorptiometry (DXA)	It correlates with low muscle mass measured by MRI, and it is cheap, quick, and widely available, with low radiation risk.	Not portable and inaccurate in patients with obesity and edema, while variations between protocols limit comparison of results.	May be used as a cheaper and quick alternative of CT. However, it should not be used in obese or edematous patients.	[98, 99, 104]

Ultrasound	Simple, cheap, safe, portable, and noninvasive, with a high reproducibility.	Its measurement lacks standardization, and it is not clear which anatomical sites can best predict total skeletal muscle mass.	Can be a safe and cheap method for frequent muscular assessment over the course of hospital/ICU stay.	[93, 94, 99, 105]

Bioelectrical impedance analysis (BIA)	It yields results concordant with CT, and it is cheap, noninvasive, and widely available.	Results are confounded by body water distribution. It reports higher SMM in patients who are males or have edema than CT. Its equations and cutoff values are population and device specific.	Not preferred in ICU patients because of changes in the hydration status.	[95, 98, 106]

Electromyography	It is a noninvasive way to assesses neuromuscular transmission denervation and deposition of endomysial connective tissue and fat.	Not widely available and requires special technical skills.	It has been used to measure maximal voluntary contraction for quadriceps and biceps in recovering patients.	[46, 99, 107]

Total/partial body potassium (TBK/PBK)	It is a cheap and simple alternative of CT and MRI with less radiation exposure. It yields results consistent with DXA.	It is based on assumptions that may not hold in old and diseased conditions, e.g., fixed muscle content of nitrogen and hydration coefficient of lean body mass.	May not accurately reflect on muscle mass because critical COVID-19 patients exhibit nitrogen loss as well as multiple micronutrient deficiencies and electrolyte imbalance.	[28, 99, 108, 109]

Anthropometric measures (body mass index and circumferences of the calf, thigh and mid-arm)	They are cheap and safe techniques that fit in low-resource facilities because they do not require special skills.	They do not accurately quantify muscle mass and may be confounded by edema and adiposity.	They detected malnutrition and wasting in ICU-admitted COVID-19 patients, especially among diabetics with cytokinemia and hypoproteinemia.	[21, 28, 87, 110]

## Data Availability

No data were used in this study.

## Abbreviations

ADL: Activities of daily living
AGEs: Advanced glycation end products
ASM: Appendicular skeletal muscle mass
AWGS: Asian Working Group for Sarcopenia
BIA: Bioelectrical impedance analysis
CFS: Clinical Frailty Scale
CK: Creatine kinase
COVID-19: Coronavirus disease 2019
CT: Computed tomography
D3Cr: D3-creatine dilution
DXA: Dual-energy X-ray absorptiometry
EMG: Electromyography
eTSMM: Total skeletal muscle mass estimated by Lee's formula
EUGMS: European Geriatric Medical Society
EWGSOP: European Working Group on Sarcopenia in Older People
FI: Frailty index
IGFs: Insulin-like growth factors
ICFSR: International Conference on Frailty and Sarcopenia research
IL: Interleukin
ICU: Intensive-care unit
LDH: Lactate dehydrogenase
LESM: Lower extremity skeletal muscle mass
MAMC: Mid-arm muscle circumference
MRI: Magnetic resonance imaging
NMJ: Neuromuscular junction
PBK: Partial body potassium
PSCA: Physiological cross-sectional area
QoL: Quality of life
RR: Risk ratio
RSMI: Relative skeletal muscle index
SMI: Skeletal muscle mass index
SNPs: Single-nucleotide polymorphisms.
